# Central Systolic Blood Pressure Is Associated With Early Vascular Damage in Children and Adolescents With Type 1 Diabetes

**DOI:** 10.3389/fcvm.2021.606103

**Published:** 2021-09-07

**Authors:** Angela Tagetti, Claudia A. Piona, Denise Marcon, Alice Giontella, Lorella Branz, Stefano Bortolotti, Anita Morandi, Claudio Maffeis, Cristiano Fava

**Affiliations:** ^1^Department of Medicine, General Medicine and Hypertension Unit, University of Verona, Verona, Italy; ^2^Pediatric Diabetes and Metabolic Disorders Unit, Regional Center for Pediatric Diabetes, University City Hospital of Verona, Verona, Italy

**Keywords:** adolescent, carotid intima-media thickness, arterial pressure, child, risk factors, type 1 diabetes, carotid-femoral pulse wave velocity

## Abstract

**Objective of the study:** This study aimed to test the effect of multiple cardiovascular risk factors on subclinical indices of atherosclerosis in children and adolescents with type 1 diabetes (T1D).

**Methods:** Carotid intima-media thickness (cIMT), carotid distensibility coefficient (cDC), and carotid–femoral pulse wave velocity (PWV) were measured in children and adolescents with T1D, in a follow-up at the outpatient clinics of Verona. Blood pressure (BP; both central and peripheral), metabolic and other cardiovascular risk factors were evaluated in multivariate linear regressions to assess the association with the measured indices of subclinical vascular damage.

**Results:** One hundred and twenty-six children and adolescents were included. cIMT was above the 95th percentile for age and height in 60.8% of the population, whereas 26% of the sample had cDC impairment (less than the 5th percentile) and 4.8% had an elevated PWV. Independent determinants of cIMT according to the regression models were only gender type of glucose monitoring and central systolic BP (cSBP). PWV was associated with age, sex, heart rate, and cSBP; cDC with age and both cSBP and, alternatively, peripheral BP (pBP). Neither pBP nor any of the tested metabolic parameters, including glycated hemoglobin, was associated with PWV and cIMT.

**Conclusions:** A high proportion of early vascular damage, especially an increased cIMT, is present in children and adolescents with T1D in whom cSBP seems to be a common determinant. In children and adolescents with T1DM, a special focus should be on hemodynamic risk factors beyond metabolic ones.

## Introduction

Ischemic heart disease and stroke due to atherosclerosis are responsible for a combined 15.2 million deaths according to the most recent WHO data, and they are the leading cause of death worldwide (1). Even if clinical manifestations occur in adulthood, atherosclerosis is a continuum process that begins early in childhood (2, 3), especially if major risk factors such as diabetes and hypertension are present. Thus, it is particularly important to address cardiovascular risk factors early in life as clearly emphasized in clinical practice guidelines also (4–6). Little is known about the association between cardiovascular and metabolic risk factors with subclinical atherosclerosis. Emerging evidence now suggests that central pressure (cSBP) is better related to future cardiovascular events than brachial pressure due to the better indication of the left ventricular load (7, 8). Hyperglycaemia effects on microvascular and macrovascular complications are well documented in patients with type 1 diabetes (T1D) (9, 10). Several studies demonstrated that T1D patients have higher common carotid intima-media thickness (cIMT) as compared with healthy volunteers since childhood (11, 12). In children with T1D, especially peripheral systolic blood pressure (pSBP) and body mass index (BMI) were related to the cIMT increase over time, underlining the importance of lifestyle and cardiovascular risk factors control (13). In the SEARCH CVD study, which evaluated 402 young patients with T1D in comparison with 206 matched controls, pSBP, sex, age, and adiposity were independently associated with cIMT, and the correlation was probably driven by the poor glycemic control (14). In a 2-year follow-up study on the same sample of T1D, children with baseline higher HbA1c and pSBP showed a steeper progression of cIMT (15). Other studies found that cIMT is not always augmented in diabetic children (16), and other markers of early atherosclerosis, such as pulse wave velocity (PWV) or carotid distensibility (cDC) could be useful if detected even earlier in childhood (17). PWV is a powerful tool for measuring the stiffness of large arteries and enables the identification of high-risk populations that might benefit from more aggressive cardiovascular disease (CVD) risk factor management (18). Finally, cDC may predict future total cardiovascular (CV) events, CV mortality, and all-cause mortality, at least in adults; measuring cDC may facilitate the identification of high-risk patients for the early diagnosis and prompt treatment of CVD (19). The present study aimed to assess the prevalence of impaired vascular indices in children and adolescents with T1D and to analyze the effect of multiple cardiovascular risk factors on diverse subclinical indices of atherosclerosis.

## Methods

### Study Population

This crosssectional study was conducted at the Regional Center for Pediatric Diabetes in collaboration with the Vascular clinic of the General Medicine and Hypertension Unit of the “University City Hospital” of Verona. The study protocol was approved by the Institutional Ethics Committee of Verona (Italy). Informed consent was obtained from the study participants and their parents.

One hundred and twenty-six children and adolescents with T1D were consecutively enrolled between March 2018 and November 2018. The inclusion criteria were an age between 10 and 20 years, and diagnosis of T1D at least 2 years before study enrolment. The exclusion criteria were as follows: having a diagnosis of diabetic microvascular complications or known cardiovascular disease according to the current ISPAD guidelines (20), and the presence of any other systemic chronic disease other than T1D, except for euthyroid Hashimoto's thyroiditis and/or celiac disease with adequate compliance to gluten-free diet.

### Clinical and Biochemical Data Collection

At the time of study enrolment, all study participants underwent a physical examination with the collection of anthropometric (height, weight, waist circumference) and peripheral blood pressure (pBP) data. BMI values were standardized calculating age and gender-specific BMI *Z*-scores and percentiles using the WHO child growth standards (21). At the end of the physical examination, pBP was measured on the left arm in a sitting position three times using a digital sphygmomanometer with a cuff appropriate for the age and arm circumference of the children. The mean of the three measurements was recorded for the analysis and pBP values were Z-score transformed according to European normative values (22). The type of insulin therapy and daily insulin dosages were recorded. Venous blood and urinary samples were collected after an overnight fast. HbA1c and other biochemical parameters [natural logarithm of triglycerides (LnTg), total cholesterol, HDL-cholesterol, LDL-cholesterol, and urinary albumin–creatinine ratio] were analyzed in a single reference centralized laboratory with standard methods. Age of onset and duration of T1D were recorded through the revision of clinical charts.

### Vascular Measurements

The cIMT (in mm) and the carotid distensibility coefficient (cDC, × 10^−3^/kPa) were measured by ultrasound (LOGIQ P5 pro, GE, Indianapolis, USA) and processed using dedicated hardware (Cardiovascular Suite, Quipu, Pisa, Italy). cIMT was measured within 1 cm from the bulb. cDC of the common carotid artery, both on the right and left sides, was taken contemporarily to the measurement of pBP using the Omron 705IT device and was calculated using the following formula: cDC = (Δ*A/A*)/*PPa*, where Δ*A* is the stroke change (i.e., the distension) in the carotid artery crosssectional area, normalized for the total diastolic carotid artery crosssectional luminal area (*A*), and *PPa* is the differential pressure, assuming that the crosssection of the artery is circular (23). cIMT and cDC values were transformed in Z-score according to the reference values proposed by Doyon et al. (24). PWV was measured using the SphygmoCor XCEL device using a cuff around the femoral artery that captures the femoral waveform and a tonometer that captures the carotid waveform. The length of the arteries was measured using a measuring tape. The velocity is computed by dividing the distance between the carotid and femoral arteries using the pulse transit time. Z-scores were computed for PWV according to the reference proposed for the applanation methods (25). cSBP was derived by the SphygmoCor XCEL device; the cuff pulsations were recorded at the brachial artery level, and then a general transfer function was applied to calculate aortic waveform. The measurement was recently validated in the pediatric population also (26). *Z*-score and percentiles were computed for cSBP also (27). Measurements were taken by a single operator who was specifically trained.

### Normative Values

For metabolic parameters, ISPAD cut-off is adopted (20, 28). Obesity and overweight status are defined according to the WHO definition (21).

### Statistical Analysis

All data were analyzed by the SPSS statistical computer package (version 21.0; IBM Corporation, Armonk, NY, USA). Continuous variables are presented as mean and standard deviation unless otherwise stated. Bivariate parametric correlations were estimated by the Pearson coefficient (*r*). Multiple linear regression analyses were used in the multivariate models with either cIMT or cDC or PWW as the dependent variable. In our model, the covariates were age at visit, sex, diabetes duration, BMI, HDL-cholesterol, LDL-cholesterol, Ln of triglycerides, HbA1c, and pBP or cSBP. *T*-test for independent samples was used to compare means in different groups. Analysis of covariance was performed using vascular measurements (cIMT, cDC, PWV, and cSBP) as dependent variables, controlling for sex and age, and testing if there was any difference in T1D patients having glycated hemoglobin higher or lower than 7.5%. All the tests were two-sided, and *P* < 0.05 were considered statistically significant.

## Results

### Sample Characteristics

Among 126 patients (mean age 16.2 ± 2.6 years) 58 were female (46%). Table 1 shows the characteristics of the sample. At the time of enrollment, insulin therapy was administered with multiple daily injections (MDI) in 104 patients (82.5%). Among them, 72 subjects (57.1%), were using continuous glucose monitoring (CGM; including intermittently scanned continuous glucose monitoring or real-time continuous glucose monitoring), whereas self-monitoring of capillary blood glucose (SMBG) was used in 54 subjects (42.9%). Twenty-two patients (17.5%) used continuous subcutaneous insulin infusion (CSII). The comparison of patients categorized according to insulin treatment (MDI vs. CSII) shows no differences in vascular indices (see Supplementary Tables 1, 2), whereas the categorization according to the type of glucose monitoring demonstrates higher cIMT in the CGM group (see Supplementary Tables 3, 4). Nobody was using cardiovascular medications.

**Table 1:** Characteristics of the 126 T1DM children and adolescents.

	**Value**	**Z-score**	**Percentiles**
*N*	126		
Female, *n* (%)	58 (46%)		
Age, years	16.2 ± 2.57		
Pre-Pubertal, *n* (%)	4 (3.2%)		
BMI, Kg/m^2^	22.02 ± 3.30	0.31 ± 0.82	59.34 ± 25.11
SBP, mmHg sitting (oscillometric)	108.6 ± 8.99	−0.52 ± 0.86	34.41 ± 24.87
DBP, mmHg sitting (oscillometric)	69.0 ± 7.36	0.13 ± 0.70	54.86 ± 23.00
cSBP, mmHg	103.3 ± 8.49	−0.15 ± 1.11^*^	44.37 ± 31.10
cIMT, mm	0.484 ± 0.08	1.95 ± 1.51^*^	85.92 ± 22.76
cDC, KPa-1 × 10^−3^	44.25 ± 10.26	−1.02 ± 0.86^*^	22.01 ± 19.70
PWV, m/s	4.70 ± 0.64	−0.79 ± 0.88^*^	26.33 ± 23.25
HbA1c, %	8.06 ± 0.93		
Total cholesterol, mmol/l	3.94 ± 0.7		
LDL-C, mmol/l	1.99 ± 0.56		
HDL-C, mmol/l	1.52 ± 0.37		
Triglycerides, mmol/l	0.76 ± 0.41		
ACR, mg/mmol of creatinine	1.50 ± 3.79		
Diabetes duration, years	6.85 ± 3.52		
Insulin dosage/Kg, U/Kg	0.89 ± 0.25		

*ACR, albumin creatinine ratio; BMI, body mass index; cDC, carotid distensibility coefficient; cIMT, carotid Intima-Media Thickness; cSBP, central Systolic Blood Pressure; DBP, diastolic Blood Pressure; HbA1c, glycated hemoglobin; HDL, high density lipoprotein; LDL, low density lipoprotein; PWV, pulse wave velocity; SBP, systolic blood pressure*.

* *z- score for sex and height*.

**Figure 1:**
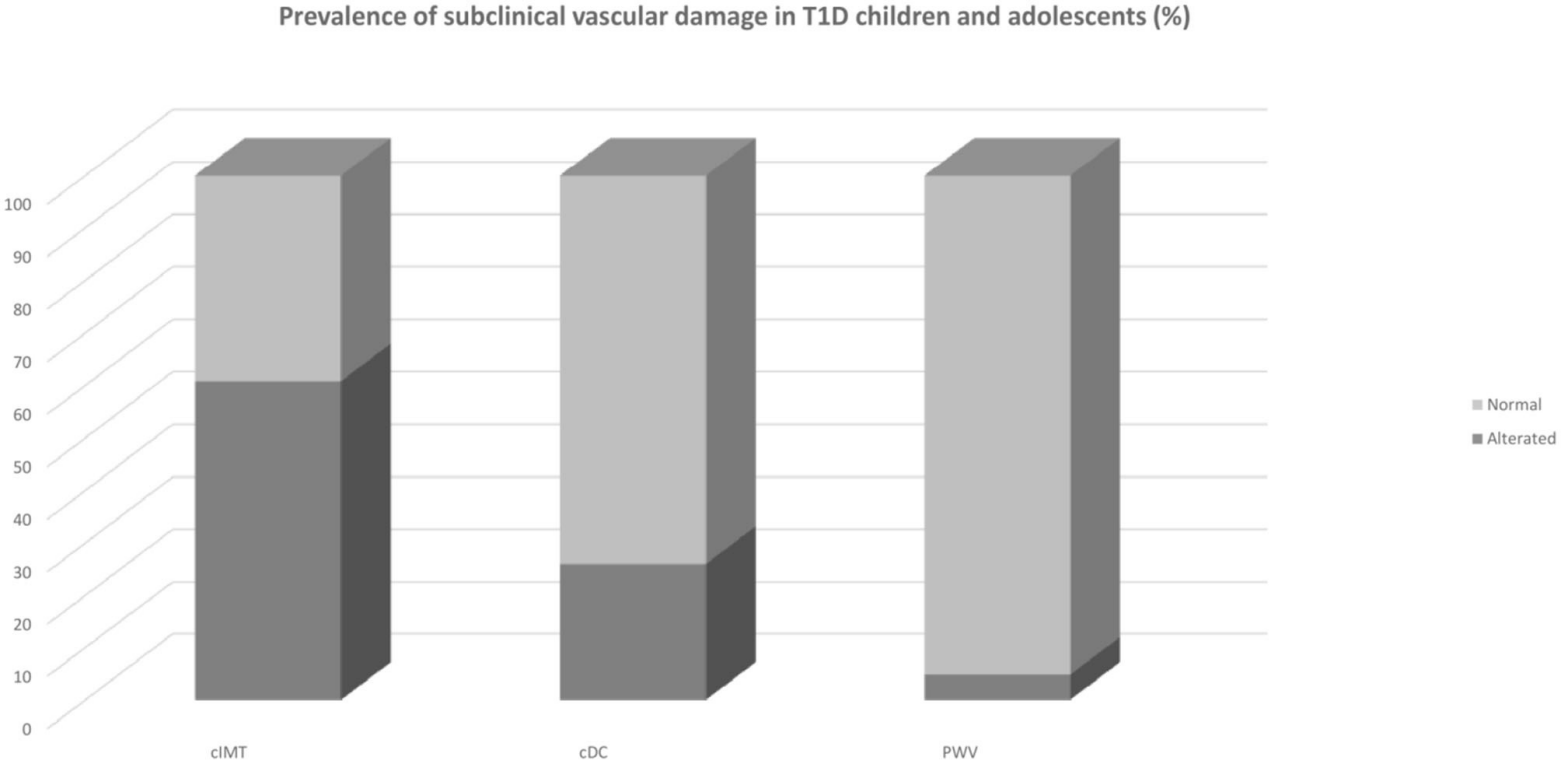
Prevalence of subclinical vascular damage in T1D children and adolescents. cDC, carotid distensibility coefficient; cIMT, carotid Intima-Media Thickness; PWV, pulse wave velocity; T1D Type 1 Diabetes.

The average duration of diabetes was 6.8 ± 3.5 years. Around 19.8% had a duration longer than 10 years for diabetes. Mean HbA1c was 8.06 ± 0.83%; LDL-cholesterol was above the recommended values in 13.7% of the population; triglycerides levels were optimal in 97% of the cases; 3.2% population were obese, and 14.3% overweight. Only one child had pBP higher than 95th percentile and only eight subjects (6.3%) had pBP in the range of prehypertension; cSBP was above the limit in 4.1% of the population. Regarding vascular measurement, cIMT was elevated in 60.8% of the population, whereas 26% of the sample had impaired cDC (<5th percentile) and 4.8% had an elevated PWV (see Figure 1). Analysis of covariance did not find any significant difference in mean cIMT [*F*_(1, 122)_ = 0.007, *p* = 0.934], cDC [*F*_(1, 119)_ = 0.0042, *p* = 0.838], PWV [*F*_(1, 119)_ = 0.028, *p* = 0.868], and cSBP [*F*_(1, 119)_ = 0.2155, *p* = 0.868] between groups having good or poor glycemic control when adjusted for age and sex. Levene's test and normality checks were carried out and the assumptions met for all the tested cases (for cIMT *p* = 0.463, for cDC *p* = 0.514, for PWV *p* = 0.531, for cSBP *p* = 0.803).

### Univariate Associations

**Figure 2:**
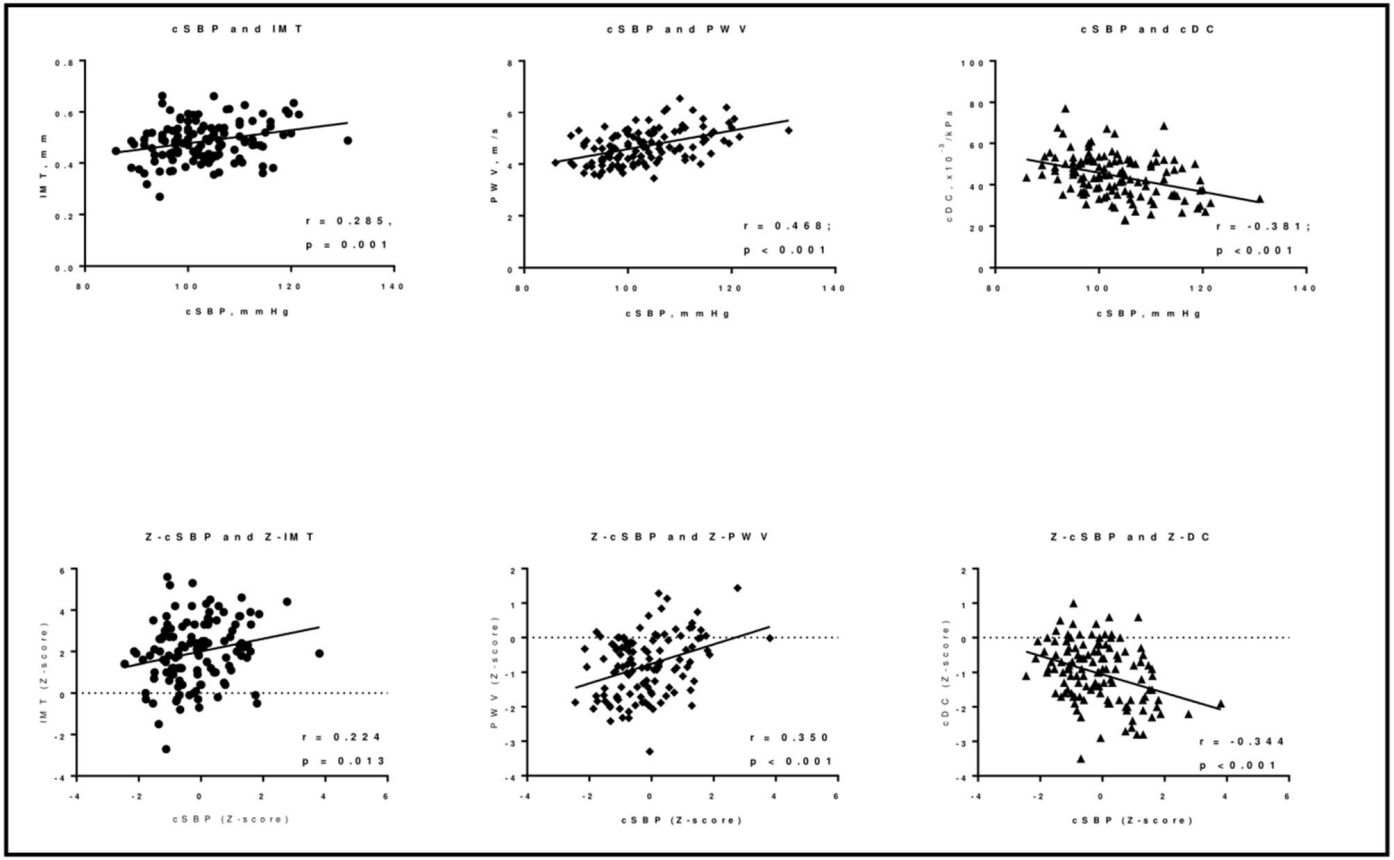
Correlations between cSBP and the indices of early vascular damage in T1D children and adolescents. cDC, carotid distensibility coefficient; IMT, carotid Intima-Media Thickness; cSBP, central Systolic Blood Pressure; PWV, pulse wave velocity. *z- score for sex and height.

Table 2 reports the correlation coefficients between vascular measurements and hemodynamic and metabolic parameters. Bivariate correlations showed cSBP, but not pBP, to be positively associated with cIMT (*r* = 0.285; *p* = 0.001). No metabolic parameters were directly associated with cIMT. Both pBP and cSBP were positively associated with PWV (for pSBP *r* = 0.307, for pDBP *r* = 0.302, for cSBP *r* = 0.468; *p* ≤ 0.001 for all) and negatively with cDC (for pSBP *r* = −0.373, for pDBP *r* = −0.385, for cSBP *r* = −0.381; *p* < 0.001 for all; see Figure 2). Regarding metabolic variables, only HDL-cholesterol was associated with both cDC (*r* = 0.183; *p* = 0.044) and PWV (*r* = −0.318; *p* < 0.001). No association was found between HbA1c and the tested markers of early atherosclerosis. When considering the correspondent *Z*-scores (for sex and height) all the correlations were confirmed except for the one between *Z*-score PWV and *Z*-pBP (Supplementary Table 5). Even a correlation between the *Z*-score of PWV and log-transformed triglycerides was evident (*r* = 0.203; *p* < 0.05). The association between BP and metabolic variables are presented in Supplementary Table 6.

**Table 2:** Correlations between vascular indices and haemodynamic/metabolic parameters.

	**cIMT**	**cDC**	**PWV**
**Blood pressure**			
pSBP	0.078	−0.373^**^	0.307^**^
pDBP	0.068	−0.385^**^	0.302^**^
cSBP	0.285^**^	−0.381^**^	0.468^**^
**Metabolic parameters**			
HbA1c	0.005	−0.028	0.017
ACR	−0.035	−0.067	0.098
Total cholesterol	0.057	0.057	0.001
HDL	−0.143	0.183^*^	−0.318^**^
LDL	0.092	0.010	0.082
LnTg	0.019	−0.071	0.151
BMI	0.062	−0.144	0.245^**^
**Other parameters**			
Diabetes duration	0.009	−0.107	0.033
Age	0.005	−0.37^**^	0.422^**^

*ACR, albumin creatinine ratio; BMI, body mass index; cDC, carotid distensibility coefficient; cIMT, carotid Intima-Media Thickness; cSBP, central Systolic Blood Pressure; HbA1c, glycated hemoglobin; HDL, high density lipoprotein; LDL, low density lipoprotein; PWV, pulse wave velocity; pSBP, peripheral systolic blood pressure*.

* *p < 0.05*,

** *p < 0.001*.

### Linear Regressions

Multiple linear regression models are presented in Table 3 and in the supplementary data (Supplementary Tables 7, 8). The only variables independently and positively associated with cIMT are gender, type of glucose monitoring and cSBP (see Table 3). For PWV, the association was with age, sex, heart rate, and cSBP. Neither influences of pBP nor metabolic parameters could be detected on PWV and cIMT. cDC was associated with age, peripheral, or alternatively cSBP values.

**Table 3:** Linear regressions of cIMT on CV risk factors.

**Dependent**	**Covariates**	**β**	**SEM**	***p*-value**	**Dependent**	**Covariates**	**β**	**SEM**	***p*-value**
cIMT	Age	−0.005	0.003	0.145	z-IMT	Age	−0.117	0.063	0.066
	Sex	0.036	0.014	0.013		Sex	0.639	0.284	0.026
	T1D duration	0.002	0.002	0.379		T1D duration	0.028	0.042	0.512
	BMI	−0.002	0.002	0.471		z-BMI	0.023	0.185	0.901
	HDL	−0.001	0.001	0.259		HDL	−0.015	0.011	0.173
	LDL	0.000	0.000	0.148		LDL	0.013	0.007	0.058
	LnTg	−0.004	0.008	0.629		LnTg	−0.109	0.154	0.480
	HbA1c	−0.002	0.008	0.746		HbA1c	−0.091	0.151	0.548
	cSBP^*^	0.003	0.001	0.006		z-cSBP^**^	0.307	0.132	0.021

* *If we substitute in the model cSBP with either pSBP or pDBP the association with pBP is not significant*.

** *If we substitute in the model Z-cSBP with either Z-pSBP or Z-pDBP the association with Z-pBP is not significant*.

*BMI, body mass index; cIMT, carotid Intima-Media Thickness; cSBP, central Systolic Blood Pressure; DBP, diastolic Blood Pressure; HbA1c, glycated hemoglobin; HDL, high density lipoprotein; LDL, low density lipoprotein; LnTg, natural logarithm of triglycerides; SBP, systolic blood pressure; T1D, Type 1 Diabetes*.

## Discussion

The most impressive result of this study is the very high frequency of subclinical vascular damage in children and adolescents with T1D as compared with the percentiles obtained in samples that collected healthy subjects, in particular, 60.8% of the participants have a cIMT greater than the 95th percentile according to age and height, cDC is lower than the 5th percentile according to sex and height in 26% of the population. For PWV, 4.8% was above the 95th percentile according to sex and height.

We have to acknowledge the lack of a control group with regard to the high prevalence of early vascular damage. Even if we chose to compare our T1D participants with samples with similar characteristics (24, 25, 27), we cannot exclude that our patients could be different in terms of ethnicity, diet, physical activity habits, or other factors with respect to these referral samples. Nevertheless, this usually happens in clinical practice, where either blood pressure or organ damage is interpreted in reference to percentiles. Moreover, our results, on average, are in line with previous studies, including a recently published meta-analysis (29). Even the average level of glycated hemoglobin was comparable with that of our population (8.2%). We would like to underline that the cIMT measurement in our study was extremely accurate; it was measured using a technology that allows us to measure digitized and real-time segment (about 1 cm of common carotid). Moreover, the population from which the percentiles were obtained, had included a relatively large sample consisting of 1,155 healthy children (24).

The link between cSBP and cIMT, but not between peripheral BP and cIMT, is the most relevant point of this study. To our knowledge, no data exist on the relationship between cSBP and cIMT in the pediatric population with diabetes. A recent Chinese study, performed in young adults, reported that cSBP, but not pSBP, is independently associated with cIMT progression. Z-cIMT was positively associated with Z-BMI, an association that was maintained after adjusting for age, sex, and cardiovascular risk factors (30). Another recent paper on children found that 24-h systolic BP, but not cSBP, is linked to Z-cIMT in the multivariate regression analysis (31). The same study found an association between cSBP and LVH in children similar to the data coming from Litwin and colleagues (32), but again the subject included in the studies were hypertensive children. High pBP is widely accepted as one of the main traditional cardiovascular risk factors. A possible explanation for the superiority of cBP over pBP in the association with early vascular damage is the fact that the estimated cSBP is closer to the BP that “challenges” the big arteries. Indeed, heart, kidneys, and carotid arteries are exposed to aortic, rather than brachial, pressure. Therefore, early cardiovascular modifications could ultimately be more related to central rather than brachial BP, as an index expressing the “real” hemodynamic stress acting on it. If these results will be confirmed and further investigated, a stricter follow-up and ABPM or home BP measurement should be warranted to identify masked hypertension.

About cDC, the results correlated with both peripheral and cSBP, but this is a well-known association partly due to the fact that pulse pressure enters the formula to calculate it.

Interestingly, no correlation was found between either carotid early damage or the duration of the disease with the glycated hemoglobin, as in a recent study by Giannopoulou et al. (29). These results are consistent with another large sample of more than 200 children with T1D (33), but not with a smaller group of T1D adolescents (34). About arterial stiffness, several studies suggest a major role of glycated hemoglobin in determining PWV, contrary to ours. Both Terlemez et al. (35) and Obermannova et al. (36) found a positive correlation between glycated hemoglobin and arterial stiffness in 70 T1D children. Even our in-depth analysis stratifying the sample according to glycated hemoglobin level did not show any significant difference in the indices of vascular damage in patients with poor versus good glycemic control. We chose to use the previously recommended ISPAD glycemic cut-off of 7.5% even if the most recent ISPAD guidelines (37) indicated a target of HbA1c of <7.0% in T1D. This is because the 7.5% cut-off (38) is still widely used in populations vulnerable to hypoglicaemia, such as children and adolescents. The reason for this lack of effect could be related to fact that there is little variability in the values of HbA1c in our cohort (in our sample 84% of the subjects with diabetes have HbA1c higher than 7%). It is well- known that time is a pivotal factor for vascular damage in T1D also that after 10 years of diabetes, CVD becomes the leading cause of death, eventually accounting for 40% of all deaths after 20 years of the disease (37). In our sample, the duration of diabetes was not associated with either index of subclinical atherosclerosis. This can be due to the relatively young age of the study participants. In our study, no other traditional cardiovascular risk factors seem to be linked with the markers of vascular damage, neither LDL cholesterol nor pBP. These results are discordant with other observations including the study by Dalla Pozza, who found cIMT to be predicted by total cholesterol beyond age at onset of the disease, insulin dosage, and pSBP (33). In other studies, in children and adolescents without diabetes, lipid levels correlate with cIMT thickening (38, 39). Thus, our findings could be related to the relatively good lipid profile of the children and adolescents followed by our center. Surprisingly, from our data, no relationship between BMI and the markers of early arterial atherosclerosis could be detected. Previous studies aimed to discriminate if the association between BMI and arterial properties was dependent or not on the exposure to other cardiovascular risk factors, which showed controversial results. Skilton et. al reported that cIMT measured at 8 years was strongly associated with weight gain, height-adjusted weight gain, and change in weight-for-height Z score from 0 to 18 months (40). Recently, a meta-analysis including 18 studies showed that childhood BMI predicted adult cIMT (41). In our sample, the prevalence of obesity and overweight is lower than in the general population (3.2 vs. 9.3% and 14 vs. 21%, respectively, according to the WHO percentiles) (21, 42). The fact that children and adolescents included in this study are strictly monitored by a nutritionist may, in part, be the reason for that. PWV was mostly in the normal range in our sample. A meta-analysis by Wang et al. reported that increased PWV in T1D patients is compatible with an increase of arterial stiffness (43). In the light of the important association with the central and peripheral pressure values, however, these findings suggest a close dependence of PWV on BP values, rather than other atherosclerotic risk factors, as expected.

Potential limitations of this study are as follows: (a) the crosssectional design of the study allows only to detect an association between variables in a static moment of a long-course condition; (b) we did not include nutritional data or information about physical activity that can play an important role in the cardiovascular system; and (c) we did not have a control group. We are aware that the lack of a control group could be a limitation. Obtaining the permission to have a control group of children in Italy is otherwise very challenging; so we decided to rely on percentiles. The study was designed considering the lack of the control group. To overcome this pitfall; the methodological and statistical tools used for obtaining these results pinpoint the relation between arterial stiffness and vascular damage *per se*. We did not want to analyze the relation implied between arterial stiffness and vascular damage. However, underlining the fact that a relation subsists in this special population at this early stage of the disease is useful both for the clinical management of the patients and as a baseline for further studies. Moreover, we always compare the data obtained in children and adolescents with normative values referring to the population with similar characteristics, but without T1D. The comparison with percentiles is frequently done in clinical practice and is widely present in literature as previously explained. We chose to use as a means of comparison percentiles built on populations who have characteristics similar to ours, and this allows us to compare children matched for sex, age, height, and ethnicity. The comparison with a wide and healthy population with same age, body size, sex, and ethnicity could not fill the gap of the lack of a control group, but gives us as a researcher and to the readers as colleagues and health workers a powerful tool to interpret the data.

We have also to underline some strengths of the study that are as follows: (a) our vascular measurements are extremely accurate; (b) children and adolescent with diabetes are well-characterized under metabolic and cardiovascular profile; (c) from our knowledge this is the first study that goes beyond the use of traditional BP measurements in the specific population of children and adolescents with T1D.

In conclusion, the results of our study provide an alarming photograph of the extension of subclinical vascular damage in children and adolescents with T1D, even if followed in an “excellence center,” when evaluated by percentiles. Between associated factors, cSBP seems to play a master role in early atherosclerotic changes of the arterial wall. The strict association of vascular damage with BP claims for a careful control of hemodynamic factors in children and adolescents with T1D.

## Data Availability Statement

The raw data supporting the conclusions of this article will be made available by the authors, without undue reservation.

## Ethics Statement

The studies involving human participants were reviewed and approved by Ethics Committee of Verona and Rovigo. Written informed consent to participate in this study was provided by the participants' legal guardian/next of kin.

## Author Contributions

AT, CP, CM, and CF conceived of the presented idea. AT, DM, LB, and SB measured vascular parameters. AT and CP wrote the manuscript with support from DM. CF and CM supervised the project. All authors discussed the results and contributed to the final manuscript.

## Conflict of Interest

The authors declare that the research was conducted in the absence of any commercial or financial relationships that could be construed as a potential conflict of interest.

## Publisher's Note

All claims expressed in this article are solely those of the authors and do not necessarily represent those of their affiliated organizations, or those of the publisher, the editors and the reviewers. Any product that may be evaluated in this article, or claim that may be made by its manufacturer, is not guaranteed or endorsed by the publisher.
