# Supplementary material for: Central Systolic Blood Pressure Is Associated With Early Vascular Damage in Children and Adolescents With Type 1 Diabetes
Source: Front Cardiovasc Med. 2021 Sep 7;8:606103. doi: 10.3389/fcvm.2021.606103 (PMC8454643; doi:10.3389/fcvm.2021.606103)
Supplement: Supplementary file 1 [file Data_Sheet_1.docx]

| **TABLE S1: Metabolic parameters in the subgroups of T1D children and adolescents divided according to the type of insulin therapy.** | | | |
| --- | --- | --- | --- |
|  | **MDI (N= 104)** | **CSII (N= 22)** | **p-value** |
| BMI, Kg/m^2^ | 22.32 ± 3.28 | 20.62 ± 3.07 | **0.026** |
| Age, years | 16.5 ± 2.42 | 15.02 ± 2.96 | **0.037** |
| HbA1c, % | 8.12 ± 0.96 | 7.79 ± 0.75 | N.S. |
| Total cholesterol, mg/dl | 151.84 ± 29.06 | 154.59 ± 23.31 | N.S. |
| LDL, mg/dl | 77.71 ± 22.44 | 76.41 ± 19.62 | N.S. |
| HDL, mg/dl | 57.69 ± 13.34 | 67.41 ± 17.35 | **0.02** |
| LnTg | 4.03 ± 0.98 | 3.92 ± 0.31 | N.S. |
| ACR, mg/mmol | 1.49 ± 4.02 | 1.56 ± 2.05 | N.S. |
| T1D duration, years | 7.26 ± 3.54 | 4.92 ± 2.74 | **0.001** |
| Insulin dosage/Kg, U/Kg | 0.91 ± 0.24 | 0.79 ± 0.24 | **0.043** |
| ACR: albumin creatinine ratio; BMI: body mass index; CSII: continuous subcutaneous insulin infusion; HbA1c: glycated haemoglobin; HDL: high density lipoprotein LDL: low density lipoprotein; LnTg: natural logarithm of triglycerides; MDI: multiple daily injections; T1D, type 1 diabetes. | | | |

| **TABLE S2: Hemodynamic and vascular parameters in the subgroups of T1D children and adolescents divided according to the type of insulin therapy.** | | | |
| --- | --- | --- | --- |
|  | **MDI (n= 104)** | **CSII (n= 22)** | **p-value** |
| SBP, mmHg | 108.91 ± 9.39 | 107.27 ± 6.85 | N.S. |
| z-SBP | -0.53 ± 0.86 | -0.46 ± 0.87 | N.S. |
| DBP, mmHg | 69.31 ± 7.56 | 67.68 ± 6.27 | N.S. |
| z-DBP | 0.12 ± 0.72 | 0.16 ± 0.63 | N.S. |
| cIMT, mm | 0.48 ± 0.07 | 0.50 ± 0.08 | N.S. |
| z-cIMT, (sex & height) | 1.89 ± 1.50 | 2.25 ± 1.56 | N.S. |
| cDC, KPa^-1^ x10^-3^ | 43.74 ± 9.81 | 46.71 ± 12.18 | N.S. |
| z-DC (sex & height) | -1.05 ± 0.86 | -0.87 ± 0.86 | N.S. |
| cSBP, mmHg | 103.71 ± 8.54 | 101.36 ± 8.15 | N.S. |
| z-cSBP (sex & height) | -0.14 ± 1.09 | -0.21 ± 1.21 | N.S. |
| PWV, m/s | 4.75 ± 0.63 | 4.50 ± 0.65 | N.S. |
| z-PWV (sex & height) | -0.75 ± 0.88 | -0.97 ± 0.88 | N.S. |
| cDC, carotid distensibility coefficient; cDBP, central Diastolic Blood Pressure; cIMT, carotid Intima-Media Thickness; cSBP, central Systolic Blood Pressure; CSII: continuous subcutaneous insulin infusion; DBP, diastolic Blood Pressure; MDI: multiple daily injections; PWV, pulse wave velocity; SBP, systolic blood pressure. | | | |

| **Table S3: Metabolic parameters in T1D children and adolescents divided according to the type of glucose monitoring** | | | |
| --- | --- | --- | --- |
|  | **SMBG (n= 54)** | **GCM (n= 72)** | **p-value** |
| BMI, Kg/m^2^ | 22.48 ± 3,62 | 21.69 ± 3.01 | N.S. |
| Age, years | 16.47 ± 2.51 | 16.97 ± 2.62 | N.S. |
| HbA1c, % | 7.95 ± 1.02 | 8.14 ± 0.86 | N.S. |
| Total cholesterol, mg/dl | 146.48 ± 27.34 | 156.76 ± 27.99 | **0.04** |
| LDL, mg/dl | 72.74 ± 19.33 | 81.01 ± 23.14 | **0.03** |
| HDL, mg/dl | 57.35 ± 13.20 | 60.96 ± 15.37 | N.S. |
| LnTg | 4.19 ± 0.39 | 3.88 ± 1.12 | **0.03** |
| ACR, mg/mmol | 6.93 ± 7.01 | 18.69 ± 44.82 | N.S. |
| T1D duration, years | 7.14 ± 3.60 | 6.63 ± 3.47 | N.S. |
| Insulin dosage/Kg, U/Kg | 0.87 ± 0.25 | 0.90 ± 0.25 | N.S. |
| cDC, carotid distensibility coefficient; cDBP, central Diastolic Blood Pressure; CGM: continuous glucose monitoring; cIMT, carotid Intima-Media Thickness; cSBP, central Systolic Blood Pressure; DBP, diastolic Blood Pressure; PWV, pulse wave velocity; SBP, systolic blood pressure; SMBG: self-monitoring blood glucose; T1D, type 1 diabetes. | | | |

| **TABLE S4: Hemodynamic and vascular parameters in T1DM children and adolescents divided according to the type of glucose monitoring** | | | |
| --- | --- | --- | --- |
|  | **SMBG (n= 54)** | **CGM (n=72)** | **p-value** |
| SBP, mmHg | 108.1 ± 9.7 | 109.0 ± 8.5 | N.S. |
| z-SBP | -0.64 ± 0.85 | -0.44 ± 0.86 | N.S. |
| DBP, mmHg | 69.3 ± 8.0 | 68.8 ± 6.9 | N.S. |
| z-DBP | 0.11 ± 0.74 | 0.15 ± 0.67 | N.S. |
| cIMT, mm | 0.466 ± 0.074 | 0.498 ± 0.075 | **0.016** |
| z-cIMT, (sex &height) | 1.56 ± 1.50 | 2.25 ± 1.46 | **0.012** |
| cDC, KPa^-1^ x10^-3^ | 43.13 ± 9.92 | 45.12 ± 10.51 | N.S. |
| z-DC (sex &height) | -1.11 ± 0.86 | -0.95 ± 0.85 | N.S. |
| cSBP, mmHg | 104.4 ± 8.8 | 102.5 ± 8.2 | N.S. |
| z-cSBP (sex &height) | -0.09± 1.17 | -0.20 ± 1.06 | N.S. |
| PWV, m/s | 4.69 ± 0.60 | 4.71 ± 0.68 | N.S. |
| z-PWV (sex &height) | -0.83 ± 0.80 | -0.75 ± 0.94 | N.S. |
| cDC, carotid distensibility coefficient; CGM: continuous glucose monitoring; cIMT, carotid Intima-Media Thickness; cSBP, central Systolic Blood Pressure; DBP, diastolic Blood Pressure; PWV, pulse wave velocity; SBP, systolic blood pressure; SMBG: self-monitoring blood glucose | | | |

| \| **TABLE S5: Correlations between vascular indices expressed as z-score for sex and age and blood pressure and metabolic parameters** \| \| \| \| \| --- \| --- \| --- \| --- \| \|  \| z-cIMT (sex & height) \| z-cDC (sex & height) \| z-PWV (sex & height) \| \| ***Blood pressure*** \| \| \| \| \| z-SBP (sex & height) \| -0.038 \| -0.285** \| 0.073 \| \| z-DBP (sex & height) \| 0.008 \| -0.256** \| 0.135* \| \| z-cSBP (sex & height) \| 0.224* \| -0.344** \| 0.350** \| \| ***Metabolic parameter*** \| \| \| \| \| HbA1c \| 0.004 \| -0.011 \| 0.017 \| \| ACR \| 0.005 \| -0.063 \| 0.196 \| \| Total cholesterol \| 0.120 \| 0.040 \| 0.031 \| \| HDL \| -0.095 \| 0.119 \| -0.167 \| \| LDL \| 0.146 \| 0.021 \| 0.072 \| \| LnTg \| -0.001 \| -0.062 \| 0.203* \| \| BMI percentile \| 0.074 \| 0.084 \| 0.041 \| \| ***Other parameters*** \| \| \| \| \| T1D duration \| -0.045 \| -0.074 \| -0.036 \| \| Age \| -0.097 \| -0.271** \| 0.239** \| \| ACR: albumin creatinine ratio; BMI: body but mass index; cDC, carotid distensibility coefficient; cIMT, carotid Intima-Media Thickness; cSBP, central Systolic Blood Pressure; DBP, diastolic Blood Pressure; HbA1c: glycated haemoglobin; HDL: high density lipoprotein; LDL: low density lipoprotein; PWV, pulse wave velocity; SBP, systolic blood pressure; T1D, type 1 diabetes  *p<0.05  **p<0.001 \| \| \| \| |
| --- | --- | --- | --- | --- | --- | --- | --- | --- | --- | --- | --- | --- | --- | --- | --- | --- | --- | --- | --- | --- | --- | --- | --- | --- | --- | --- | --- | --- | --- | --- | --- | --- | --- | --- | --- | --- | --- | --- | --- | --- | --- | --- | --- | --- | --- | --- | --- | --- | --- | --- | --- | --- | --- | --- | --- | --- | --- | --- | --- | --- | --- | --- | --- | --- | --- | --- | --- | --- | --- | --- | --- | --- |

| **TABLE S6: Bivariate correlation between blood pressure and metabolic parameters and between blood pressure expressed as Z-score and metabolic parameters.** | | | | |
| --- | --- | --- | --- | --- |
|  | SBP | DBP | cSBP | cDBP |
| HbA1c | -0.041 | -0.077 | -0.038 | -0.037 |
| ACR | -0.074 | -0.071 | -0.052 | -0.033 |
| Total cholesterol | -0.091 | -0.045 | -0.024 | 0.025 |
| HDL | -0.160 | -0.129 | -0.252** | -0.086 |
| LDL | -0.028 | 0.016 | 0.064 | 0.043 |
| LnTg | -0.132 | 0.015 | 0.189* | 0.133 |
| BMI | 0.380** | 0.233** | 0.413** | 0.215* |
| T1D duration | 0.114 | 0.075 | 0.054 | 0.040 |
| Age | 0.351** | 0.183 | 0.297** | 0.170 |
|  | | | | |
|  | z-SBP (sex & height) | z-DBP (sex & height) | z-cSBP (sex & height) | |
| HbA1c | 0.028 | -0.024 | -0.024 | |
| ACR | 0.099 | 0.072 | 0.059 | |
| Total cholesterol | 0.099 | 0.105 | 0.100 | |
| HDL | 0.110 | 0.099 | -0.007 | |
| LDL | 0.058 | 0.080 | 0.102 | |
| LnTg | -0.095 | 0.050 | 0.159 | |
| BMI percentile | 0.245** | 0.166 | 0.274** | |
| T1D duration | -0.040 | -0.058 | -0.101 | |
| Age | -0.107 | -0.251** | -0.019 | |
| ACR: albumin creatinine ratio; BMI: body mass index; cDC, carotid distensibility coefficient; cIMT, carotid Intima-Media Thickness; cSBP, central Systolic Blood Pressure; DBP, diastolic Blood Pressure; HbA1c: glycated haemoglobin; HDL: high density lipoprotein; LDL: low density lipoprotein; PWV, pulse wave velocity; SBP, systolic blood pressure; T1D, type 1 diabetes  *p<0.05  **p<0.001 | | | | |

| **TABLE S7: Linear regressions of cDC on CV risk factors** | | | | | | | | | |
| --- | --- | --- | --- | --- | --- | --- | --- | --- | --- |
| **Dependent** | **Covariates** | **β** | **SEM** | **p-value** | **Dependent** | **Covariates** | **β** | **SEM** | **p-value** |
| cDC | Age | -1.255 | 0.406 | 0.003 | z-cDC | Age | -1.351 | 0.397 | 0.001 |
|  | Sex | 0.529 | 1.826 | 0.772 |  | Sex | -0.286 | 1.778 | 0.873 |
|  | T1D duration | 0.052 | 0.271 | 0.849 |  | T1D duration | 0.000 | 0.266 | 0.999 |
|  | BMI | 0.310 | 0.318 | 0.331 |  | z-BMI | 1.383 | 1.164 | 0.237 |
|  | HDL | 0.015 | 0.069 | 0.827 |  | HDL | 0.039 | 0.067 | 0.557 |
|  | LDL | 0.007 | 0.043 | 0.862 |  | LDL | 0.006 | 0.042 | 0.885 |
|  | LnTg | -0.465 | 0.988 | 0.638 |  | LnTg | -0.679 | 0.962 | 0.482 |
|  | HbA1c | -0.424 | 0.971 | 0.663 |  | HbA1c | -0.376 | 0.951 | 0.694 |
|  | **cSBP*** | -0.380 | 0.118 | 0.002 |  | **z-cSBP**** | -3.101 | 0.822 | <0.001 |
| *If we substitute in the model cSBP with either pSBP or pDBP the association remains significant. **If we substitute in the model Z-cSBP with either Z-pSBP or Z-pDBP the association remains significant. | | | | | | | | | |
| BMI: body mass index; cDC, carotid distensibility coefficient; cSBP, central Systolic Blood Pressure; DBP, diastolic Blood Pressure; HbA1c: glycated haemoglobin; HDL: high density lipoprotein; LDL: low density lipoprotein; LnTg: natural logarithm of triglycerides; SBP, systolic blood pressure; T1D, type 1 diabetes | | | | | | | | | |

| **TABLE S8: Linear regressions of PWV on CV risk factors** | | | | | | | | | |
| --- | --- | --- | --- | --- | --- | --- | --- | --- | --- |
| **Dependent** | **Covariates** | **β** | **SEM** | **p-value** | **Dependent** | **Covariates** | **β** | **SEM** | **p-value** |
| PWV | Age | 0.087 | 0.026 | 0.001 | z-PWV | Age | 0.115 | 0.035 | <0.002 |
|  | Sex | 0.302 | 0.114 | 0.010 |  | Sex | 0.392 | 0.169 | 0.023 |
|  | HR | 0.015 | 0.005 | 0.003 |  | HR | 0.025 | 0.008 | 0.001 |
|  | T1D duration | -0.013 | 0.016 | 0.410 |  | T1D duration | -0.024 | 0.024 | 0.323 |
|  | BMI | 0.002 | 0.019 | 0.925 |  | z-BMI | -0.001 | 0.108 | 0.995 |
|  | HDL | -0.003 | 0.004 | 0.513 |  | HDL | 0.003 | 0.006 | 0.573 |
|  | LDL | 0.004 | 0.003 | 0.123 |  | LDL | 0.005 | 0.004 | 0.219 |
|  | LnTg | 0.048 | 0.055 | 0.386 |  | LnTg | 0.126 | 0.086 | 0.147 |
|  | HbA1c | 0.026 | 0.061 | 0.671 |  | HbA1c | 0.068 | 0.089 | 0.448 |
|  | **cSBP*** | 0.024 | 0.008 | 0.002 |  | **z-cSBP**** | 0.240 | 0.085 | 0.006 |
| *If we substitute in the model cSBP with either pSBP or pDBP the association with pBP is not significant. **If we substitute in the model Z-cSBP with either Z-pSBP or Z-pDBP the association with Z-pBP is not significant. | | | | | | | | | |
| BMI: body mass index; cDBP, central Diastolic Blood Pressure; cSBP, central Systolic Blood Pressure; DBP, diastolic Blood Pressure; HbA1c: glycated haemoglobin; HDL: high density lipoprotein; LDL: low density lipoprotein; LnTg: natural logarithm of triglycerides; PWV, pulse wave velocity; SBP, systolic blood pressure; T1D, type 1 diabetes. | | | | | | | | | |
